# A MutSβ-Dependent Contribution of MutSα to Repeat Expansions in Fragile X Premutation Mice?

**DOI:** 10.1371/journal.pgen.1006190

**Published:** 2016-07-18

**Authors:** Xiao-Nan Zhao, Rachel Lokanga, Kimaada Allette, Inbal Gazy, Di Wu, Karen Usdin

**Affiliations:** 1 Section on Gene Structure and Disease, Laboratory of Cell and Molecular Biology, National Institute of Diabetes, Digestive and Kidney Diseases, National Institutes of Health, Bethesda, Maryland, United States of America; 2 Division of Medical Biochemistry, University of Cape Town Medical School, Cape Town, South Africa; 3 Section on Physical Biochemistry, Laboratory of Biochemistry and Genetics, National Institute of Diabetes, Digestive and Kidney Diseases. National Institutes of Health, Bethesda, Maryland, United States of America; The Hospital for Sick Children and University of Toronto, CANADA

## Abstract

The fragile X-related disorders result from expansion of a CGG/CCG microsatellite in the 5’ UTR of the *FMR1* gene. We have previously demonstrated that the MSH2/MSH3 complex, MutSβ, that is important for mismatch repair, is essential for almost all expansions in a mouse model of these disorders. Here we show that the MSH2/MSH6 complex, MutSα also contributes to the production of both germ line and somatic expansions as evidenced by the reduction in the number of expansions observed in *Msh6*^*-/-*^ mice. This effect is not mediated via an indirect effect of the loss of MSH6 on the level of MSH3. However, since MutSβ is required for 98% of germ line expansions and almost all somatic ones, MutSα is apparently not able to efficiently substitute for MutSβ in the expansion process. Using purified human proteins we demonstrate that MutSα, like MutSβ, binds to substrates with loop-outs of the repeats and increases the thermal stability of the structures that they form. We also show that MutSα facilitates binding of MutSβ to these loop-outs. These data suggest possible models for the contribution of MutSα to repeat expansion. In addition, we show that unlike MutSβ, MutSα may also act to protect against repeat contractions in the *Fmr1* gene.

## Author Summary

The repeat expansion diseases are a group of human genetic disorders that are caused by expansion of a specific microsatellite in a single affected gene. How this expansion occurs is unknown, but previous work in various models for different diseases in the group, including the fragile X-related disorders (FXDs), has implicated the mismatch repair complex MutSβ in the process. With the exception of somatic expansion in Friedreich ataxia, MutSα has not been reported to contribute to generation of expansions in other disease models. Here we show that MutSα does in fact play a role in both germ line and somatic expansions in a mouse model of the FXDs since the expansion frequency is significantly reduced in *Msh6*^*-/-*^ mice. However, since we have previously shown that loss of MutSβ eliminates almost all expansions, MutSα is apparently not able to fully substitute for MutSβ in the expansion process. We also show here that MutSα increases the stability of the structures formed by the fragile X repeats that are thought to be the substrates for expansion and promotes binding of MutSβ to the repeats. This, together with our genetic data, suggests possible models for how MutSα and MutSβ, could co-operate to generate repeat expansions in the FXDs.

## Introduction

The fragile X (FX)-related disorders (FXDs) are repeat expansion diseases that result from an increase in the length of a CGG/CCG-repeat tract in the 5’ UTR of the *FMR1* gene (reviewed in [1]). This expansion occurs from an unstable premutation (PM) allele that contains 55–200 repeats. The repeat is prone to expansion in germ line and somatic cells in humans and in a FXD mouse model with a targeted insertion of ~130 FX-repeats [2–4]. The molecular basis of this instability is not known. Individual strands of the FX repeat form hairpins and other atypical structures some of which may be folded and include mismatches [5–12] and current thinking is that these structures are the substrates for the expansion pathway [13].

We have previously shown that a number of different pathways that affect repeat instability are active in a mouse model of the FXDs, one that gives rise to expansions, one that results in the error-free repair of the expansion substrate and perhaps two different contraction pathways [14, 15]. We have also shown that the mismatch repair (MMR) complex MutSβ, a heterodimer of MSH2 and MSH3, is required for 98% of germ line and all somatic expansions in the FXD mouse [14]. This is consistent with what has been seen in some, but not all mouse models of other repeat expansion diseases [16–18]. MutSα, the other MSH2-containing complex present in mammals, has been shown to either have no effect or to protect against repeat instability in various mouse models [16, 17, 19]. For example, in a mouse model for myotonic dystrophy type 1 (DM1), MutSα protects against somatic expansions [19] and in a mouse model for Friedreich ataxia (FRDA) MutSα protects against both germ line expansions and contractions [17]. However, FRDA is also unique amongst the repeat expansion diseases studied thus far in that MutSα has also been shown to be involved in generating somatic expansions in the mouse model and in patient-derived induced pluripotent stem cells [20]. Whether or not this involvement of MutSα reflects some unique property of the GAA/TTC-repeats is not known. Furthermore, why MutSα protects against, rather than promotes, germ line expansions is also an open question [17].

As part of an effort to better understand the mechanisms of repeat instability in the FXD mouse model, we examined the somatic and intergenerational instability of the FX repeat in mice lacking MSH6, the MSH2-binding protein in the MutSα heterodimer. These data, together with our biochemical studies on the binding of these complexes to CGG- and CCG-repeats have interesting implications for the mechanism of repeat instability in the FXDs.

## Results

### Loss of MSH6 reduces the extent of somatic expansions.

Our previous work indicates that in the FXD mouse model expansion, contraction and error-free pathways co-exist in germ line cells [4]. This can complicate the interpretation of intergenerational transmission data since a decrease in expansions can result either because the expansion pathway has become less efficient or because a pathway that protects against contractions has been impaired, or some combination of both. However, the interpretation of somatic instability data is simpler since all evidence to date suggests that the contraction pathway is not active in adult somatic cells in this mouse model [3, 4, 15].

**Fig 1:**
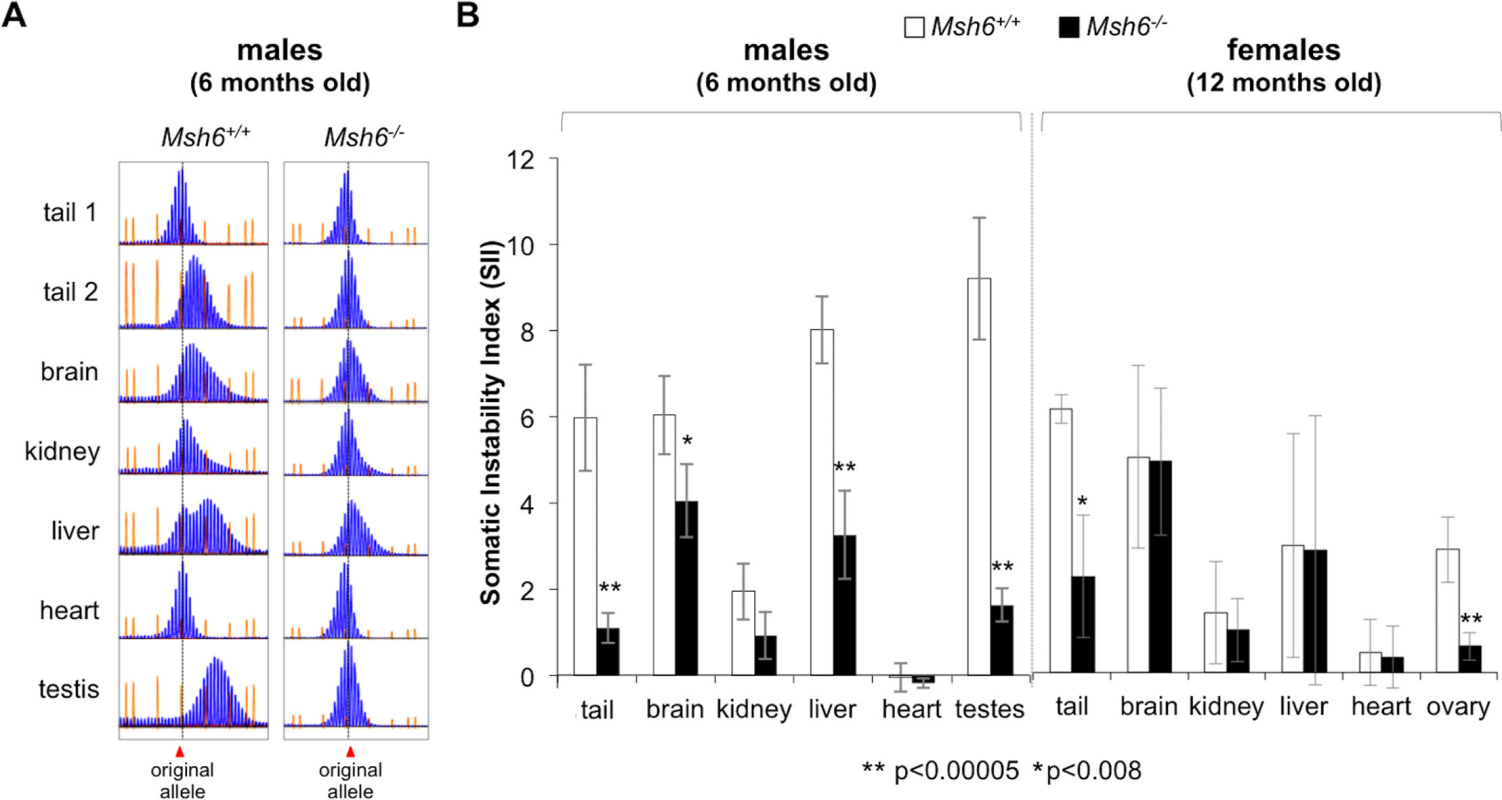
Loss of MSH6 reduces somatic expansions in both males and females. A) Representative examples of the GeneMapper profiles for the PM allele in different organs of 6 month old *Msh6*^*+/+*^ and *Msh6*^*-/-*^ males. Tail 1 refers to the tail DNA at 3 weeks of age, while tail 2 refers to the tail sample taken at 6 months of age. B) The SII for the organs of six 6-month old *Msh6*^*-/-*^ males and six *Msh6*^*+/+*^ littermates and three 12 month old *Msh6*^*-/-*^ females and four *Msh6*^*+/+*^ littermates was determined and the average SII for each organ plotted. The initial repeat number in these mice was ~160. The negative value for the heart in animals of each genotype and for the remaining organs in *Msh6*^*-/-*^ mice reflects the strand-slippage or stutter products that are typically seen when large repeat tracts are amplified rather than contraction events. Note that despite the fact that the females are twice as old as the males, the SIIs for all organs in females were either similar to or lower than they were in males. The error bars represent standard deviations.

Since somatic expansion is much more extensive in males than in females in the FXD mouse model [21], we examined the effect of the loss of MSH6 on expansion in different organs of male mice that were 6 months old. This time point was chosen since *Msh6*^*-/-*^ male mice rarely survive beyond this age. However, since somatic expansion is clearly discernable in *Msh6*^*+/+*^ males at this age, any effect of the loss of MSH6 can be readily detected. To examine the effect of the loss of MSH6 on somatic instability in male mice we carried out PCR across the repeat and then resolved the PCR products by high-resolution capillary electrophoresis. Analysis of the PCR products produced from somatic tissue of *Msh6*^*+/+*^ animals at weaning at 3 weeks of age (tail 1) or in organs that do not show somatic expansion, like heart, typically reveal a Gaussian distribution of PCR products with relatively little deviation of these products from the mean (Fig 1A).

These PCR profiles are indistinguishable from those obtained from samples taken at birth [3]. Some of these products represent strand-slippage products that are generated when amplifying through long repeat tracts. In particular, the PCR products smaller than the major allele that do not change with genotype, age or tissue, fall into this category. We then used these PCR profiles to determine the somatic instability index (SII), a quantitative measure of the extent of repeat expansion [22]. In *MSH6*^*+/+*^ males the SII for heart was -0.1 (Fig 1B). This negative number is not evidence of contractions since the SII in heart does not change with age and the PCR profile seen in old animals corresponds to the original allele size determined at birth ([3] and Fig 1A). The negative value likely reflects contribution of the products of strand-slippage to the SII. In organs other than the heart, the SII was positive with the lowest SII being seen in kidney and the highest in liver and testes as we had previously observed [3].

The loss of MSH6 was associated with a significant reduction in the SII in many organs of male mice (Fig 1B). The organs most affected are those with the highest level of expansion in *Msh6*^*+/+*^ animals, namely, the tail, brain, liver and testis. However, the distribution of products smaller than the major allele are similar in all tissues including the tail sample taken at weaning and the heart, an organ that shows little, if any instability (Fig 1A). We also did not see evidence of somatic contractions in *Msh2*^-/-^ mice that lack both MutSα and MutSβ [23]. Thus the failure to see evidence of contractions in *Msh6*^*-/-*^ mice is not the result of an offsetting effect of MutSβ-mediated expansions. We can therefore conclude that the reduced SII in *Msh6*^*-/-*^ mice is not the result of contractions that have now become apparent as a result of the loss of MSH6. Rather the reduction must reflect either a direct or indirect role of MSH6, and thus MutSα, in generating somatic expansions.

Female *Msh6*^*+/+*^ mice show less somatic expansion than males [21]. This makes it difficult to see significant effects of the loss of MSH6 in young animals. We thus confined our examination of somatic instability in *Msh6*^*-/-*^ females to the few that survived to 12 months of age. Note that despite the females being twice as old as the males, the SII in *Msh6*^*+/+*^ females was still lower than it was in most of the corresponding organs of 6 month old males, consistent with reduced somatic instability in females (Fig 1B). Nonetheless, a role for MSH6 in generating somatic expansions was apparent in *Msh6*^*-/-*^ females albeit only in tail and ovary (Fig 1B). Thus, the loss of MSH6, like the loss of MSH3, reduces the extent of somatic expansion. However, while somatic expansions are completely eliminated in *Msh3*^*-/-*^ males and females on a similar genetic background [14], some expansion is still evident in *Msh6*^*-/-*^ mice of both sexes.

### Loss of Msh6 also significantly reduces the frequency of germ line expansions

**Fig 2:**
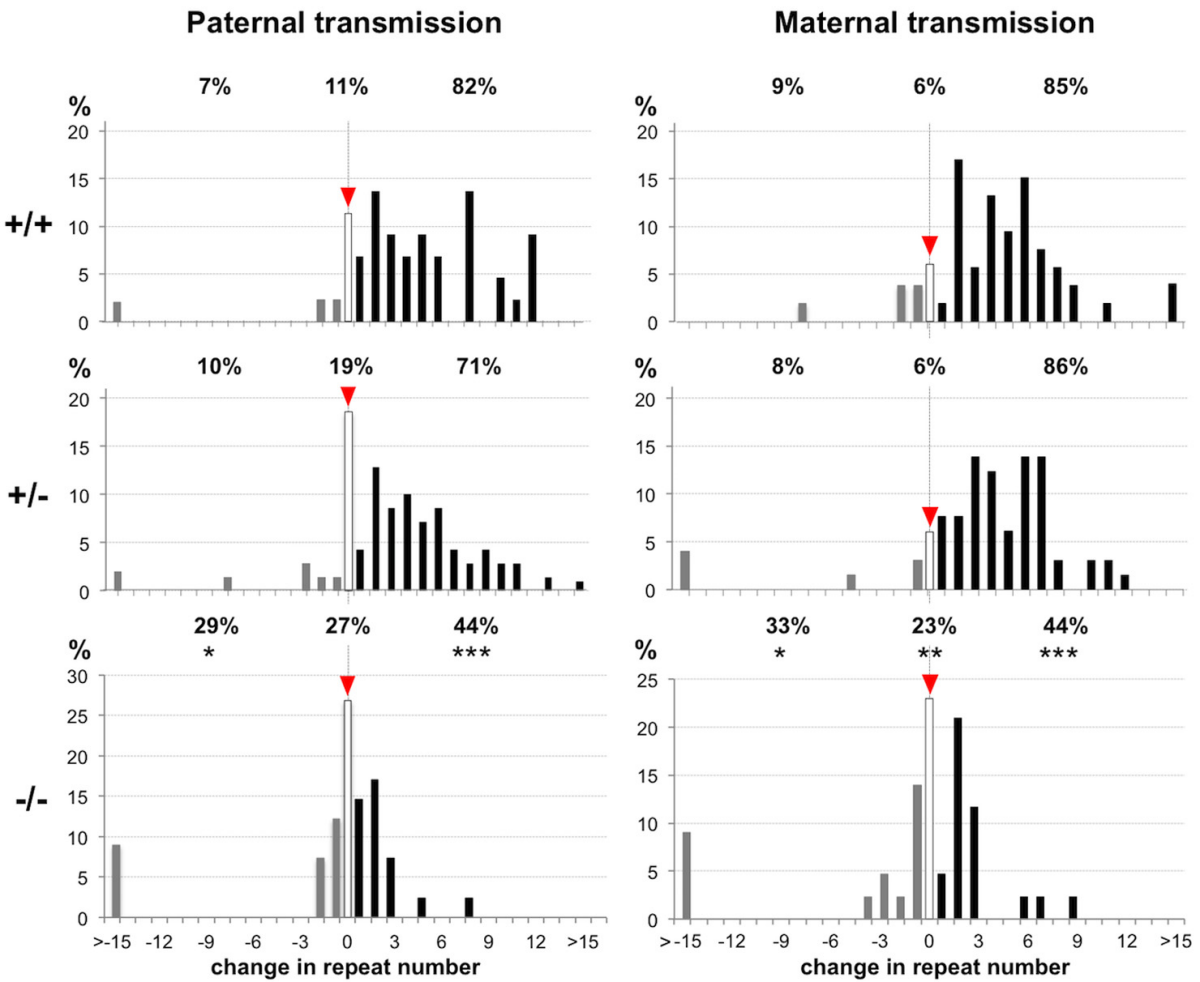
Loss of MSH6 reduces the frequency of paternally and maternally transmitted expansions. The % of alleles with the indicated number of repeats added or lost seen on transmission of a PM allele with ~160 repeats from *Msh6*^*+/+*^, *Msh6*^+/-^ and *Msh6*^*-/-*^ parents. A total of 44 *Msh6*^*+/+*^, 70 Msh6^+/-^ and 41 *Msh6*^*-/-*^ paternal transmissions and 53 *Msh6*^*+/+*^, 65 *Msh6*^+/-^ and 43 *Msh6*^*-/-*^ maternal transmissions were examined. Differences in frequency of expansions, contractions and unchanged alleles were compared for each pair of genotypes using Fisher’s exact test in which each allele class was considered in relation to the sum of the other two allele size classes. The allele classes that are significantly different from *Msh6*^*+/+*^ animals are marked with asterisks (*, p<0.05; **, p<0.01; ***, p<0.001); frequencies were also compared across all 3 genotypes with exact Jonckheere-Terpstra tests (p<0.001 for each sex).

We hypothesized that the loss of MSH6 would also affect germ line expansions with loss of two copies of the gene having a larger effect than the loss of one copy. We thus examined the transmission of the PM allele on intergenerational transfer from *Msh6*^*+/+*^, *Msh6*^*+/-*^ and *Msh6*^*-/-*^ parents. The Jonckheere-Terpstra test for ordered alternatives showed that there was a statistically significant trend towards fewer expansions with decreasing *Msh6* gene dosage (p<0.001 for both paternal and maternal transmission). Pairwise comparisons demonstrated that while the expansion frequencies in the offspring of *Msh6*^*+/-*^ parents was not significantly different from the expansion frequency in the offspring of *Msh6*^*+/+*^ parents, the progeny of *Msh6*^*-/-*^ males and females had significantly fewer expansions than the progeny of either the *Msh6*^*+/+*^ (Fisher’s exact test; p = 0.0003 for paternal and p<0.0001 for maternal transmission respectively; Fig 2) or the *Msh6*^*+/-*^ parents (Fisher’s exact test; p = 0.008 paternal and p<0.0001 for maternal transmission respectively; Fig 2).

There was also a significant difference in the distribution of the transmitted alleles for both maternal and paternal transmissions (Mann-Whitney *U* test; p<0.0001 for both males and females). There is no evidence to date to suggest that somatic and germ line expansions occur by different mechanisms in the FXD mouse. Thus the simplest interpretation of our data is that the decline in germ line expansions seen in *Msh6*^*-/-*^ animals reflects a contribution of MutSα to the germ line expansion process. This would be above and beyond the 2% of expansions that are MSH2-dependent, but MSH3-independent that we previously attributed to MutSα [14]. However, in contrast to the 80:20 ratio of unchanged to contracted alleles seen in *Msh3*^*-/-*^ males [14], in *Msh6*^*-/-*^ males the ratio was 50:50 (27% vs 29% of the total alleles). The ratio of unchanged to contracted alleles in *Msh2*^-/-^ mice is intermediate between the two (60:40) consistent with the combined contribution of MutSβ and MutSα complexes to the overall distribution of residual alleles [23]. The decline in the proportion of unchanged alleles in *Msh2*^-/-^ and *Msh6*^*-/-*^ animals relative to *Msh3*^*-/-*^ mice may reflect an additional role for MutSα in protecting against contractions which occur in the germ line, but not somatic cells. Thus the decline in expansions seen on intergenerational transmission in *Msh6*^*-/-*^ mice may represent some combination of the reduced efficacy of the expansion pathway together with the reduced efficacy of the pathway that protects against contractions.

### Loss of MSH6 does not eliminate large contractions

We have previously shown that loss of MSH3 results in a change in the distribution of contraction sizes that are seen on intergenerational transmission [14]. Specifically while animals wildtype with respect to mismatch repair show a bimodal distribution of repeat sizes with the first modal class having lost 1–2 repeats and a second modal class having lost >7 repeats, *Msh3*^*-/-*^ mice show a significant loss of alleles falling into the second modal class. Most notably in *Msh3*^*-/-*^ males all contractions involved the loss of just a single repeat. This would be consistent with a role for MutSβ in generating larger contractions.

**Fig 3:**
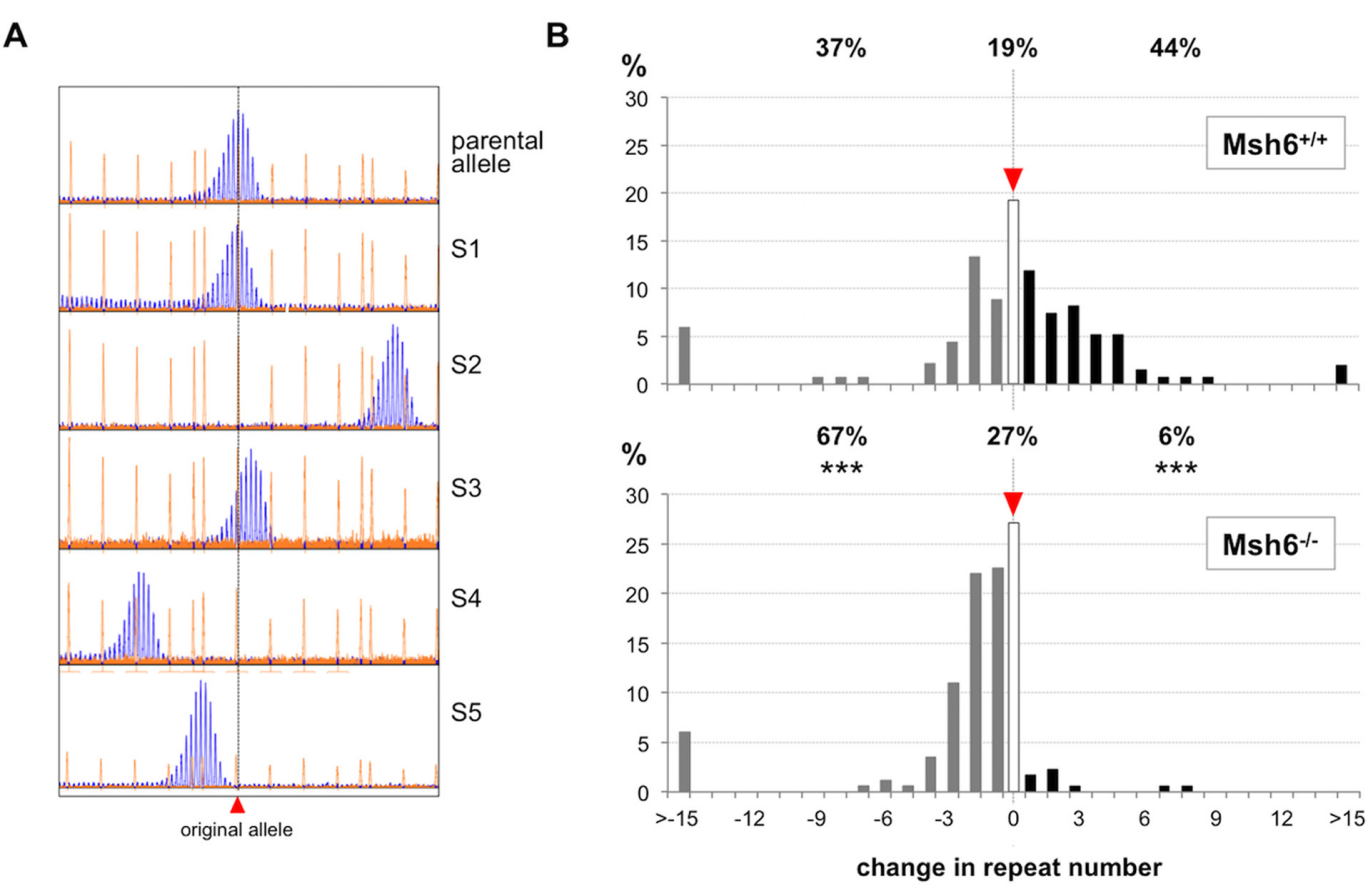
Loss of MSH6 reduces the frequency of expansions but does not significantly reduce the frequency of large contractions in the sperm of *Msh6*^*-/-*^ males. Small pool PCR was carried out on the sperm of 2-month old males as described in the Materials and Methods. A) Representative GeneMapper profiles for sperm samples analyzed by small-pool PCR showing the original paternal allele and 5 alleles, labeled S1-S5, found in sperm. B) The % of alleles with the loss or gain of different repeat numbers seen in the sperm of *Msh6*^*+/+*^ and *Msh6*^*-/-*^ mice. A total of 135 and 173 alleles were examined in the sperm of *Msh6*^*+/+*^ and *Msh6*^*-/-*^ mice respectively. The proportion of contractions, expansions and alleles that are the same size as the parental allele are indicated above each graph. Differences between these allele classes were compared for each pair of genotypes using Fisher’s exact test in which each allele class was considered in relation to the sum of the other two allele size classes. The allele classes that are significantly different from *Msh6*^*+/+*^ animals are marked with asterisks (p<0.0001). Note that the proportion of the different allele classes differs from the data shown in Fig 2. This difference is likely due to the differences in the ages of the animals in the two data sets since in males the number of expansions increases with age, although it is also possible that this reflects, in part, the contribution of other cell types that might be present in the sperm sample. Note that, in contrast to what is seen in *Msh3*^*-/-*^ animals, the distributions of the contracted alleles for both genotypes significantly deviated from unimodality (Hartigans’ dip test: p < 2.2e-16).

To assess the contribution of MutSα to contractions we examined the distribution of contracted alleles in *Msh6*^*-/-*^ animals. *Msh6*^*-/-*^ males die young making it difficult to collect enough data on the contraction sizes of paternally transmitted alleles. Therefore we analyzed the effect of the loss of MSH6 on the distribution of contraction sizes by doing small pool PCR on sperm DNA isolated from 2 month old *Msh6*^*-/-*^ males (Fig 3A). The expansion frequency in *Msh6*^*+/+*^ sperm was generally lower than that observed in the live born progeny of *Msh6*^*+/+*^ animals. This could reflect the difference in the ages of the sperm donors (2 months) versus the fathers (2–6 months), since there is a progressive increase in the proportion of expanded alleles with age [4]. A contribution of low level of contamination of the sperm sample with less expansion-prone somatic cells also cannot be completely excluded. However, the expansion frequency was also lower in the sperm of *Msh6*^*-/-*^ mice than in the progeny of *Msh6*^*-/-*^ males. Thus, as expected, there were fewer expansions and more contractions than in the sperm of *Msh6*^*+/+*^ males of the same age (Fig 3B, Fisher’s exact test; p<0.0001). In addition, the distribution of alleles in *Msh6*^*-/-*^ and *Msh6*^*+/+*^ gametes was significantly different by the Mann-Whitney *U* test (p<0.0001). This is generally consistent with the data derived from analysis of the progeny of *Msh6*^*-/-*^ males (Fig 2).

In any event, in contrast to what is seen in *Msh3*^*-/-*^ animals, the distribution of contractions in *Msh6*^*-/-*^ sperm was similar to that seen in *Msh6*^*+/+*^ sperm (Mann-Whitney *U* test; p = 0.14). Our data thus suggest that MSH6, and therefore MutSα does not severely impact the distribution of contraction sizes as does MutSβ [14].

### Loss of MSH6 does not lead to decreased levels of MSH3

**Fig 4:**
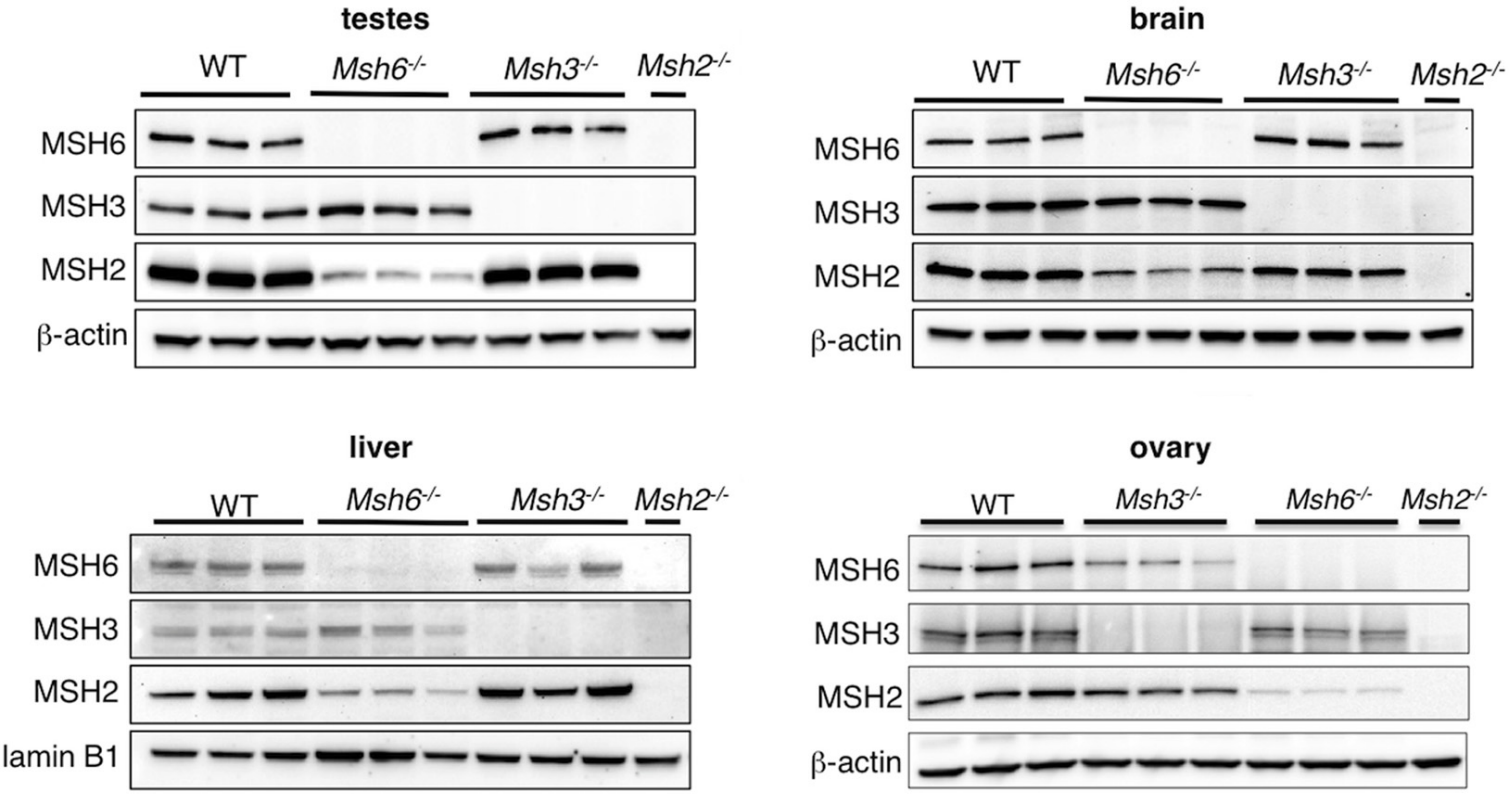
The loss of MSH6 does not affect the levels of MSH3 in brain and testes. Equivalent amounts of total protein from brain, testes and ovary and nuclear extracts from liver of three 6-month old WT, *Msh3*^*-/-*^ and *Msh6*^*-/-*^ and *Msh2*^*-/-*^ mice were subjected to electrophoresis and western blotting using the MSH2, MSH3 and MSH6 antibodies described in the Materials and Methods. Nuclear extracts of liver were used to enrich for the MMR proteins that are particularly low in this organ, and to remove a non-specific protein that cross-reacts with the MSH6 antibody. Note that the gel used for nuclear extracts (4–12% Tris-Bis) was different from the gels used to resolve total protein extracts (3–8% Tris-acetate). A representative example of a full blot showing the binding of antibodies to MSH2, MSH3, MSH6 is shown in S1 Fig. Note that in ovary and liver more than one MSH3-species was seen in both WT and *Msh6*^-/-^ animals. This most likely reflects proteolytic degradation of MSH3 during preparation of the protein extracts.

It has been suggested that MSH2 partitions between available pools of MSH3 and MSH6 and thus that the loss of MSH6 should thus not lead to a decrease in MutSβ [24–26]. To verify this we compared the levels of each of the three proteins in various organs of *Msh2*^-/-^, *Msh3*^-/-^, *Msh6*^*-/-*^ mice and mice WT for all three proteins. As can be seen in Fig 4, the absence of MSH2 led to a complete loss of bands with the predicted mobility of MSH3 and MSH6, consistent with previous data demonstrating that the formation of MutSα and MutSβ complexes protects their subunits from degradation [24, 26, 27]. The loss of MSH6 resulted in a much larger decrease in the levels of MSH2 than did the loss of MSH3 in all organs tested. However, as can be seen in Fig 4, the levels of MSH3 are comparable in the brain and testes of *Msh6*^*+/+*^ and *Msh6*^*-/-*^ mice and after normalizing to β-actin, no significant difference in the levels of MSH3 were detected. Two MSH3 bands were seen the liver and ovary of *Msh6*^*+/+*^ and Msh6^-/-^ animals that were absent in extracts from *Msh3*^*-/-*^ animals. These bands are thus likely to be MSH3-related. A similar pair of bands was seen in mouse spleen extracts using a MSH3 antibody that was directed to a similar region of the protein as the antibody we used [28]. However, in that report, only one band was detected with an antibody that recognizes a very N-terminal epitope. The N-terminal end of MSH3 known to be prone to degradation [29, 30] and it is possible that the smaller of the two bands represents a proteolytic degradation product of MSH3 in which the N-terminus had been lost. Thus, while the original levels of MSH3 in these organs are difficult to determine unequivocally, the data from brain and testes suggests that the effect of the loss of MSH6 on somatic and germ line expansion is not due to an indirect effect on the levels of MSH3, at least in some of the most expansion-prone organs in these animals.

### MutSα binds to and increases the stability of the secondary structures formed by the CCG and CGG loop-outs

We have previously shown that MutSβ binds to loop-outs formed by CCG- and CGG-repeats [14]. To see whether the same was true of MutSα, we examined the binding of this protein to substrates containing a loop-out of (CCG)_13_ or (CGG)_13._ These substrates were modeled on those used previously to examine MutSβ binding to CAG-repeats [31]. We also included MutSβ with a deletion of the unstructured N-terminal region of MSH3 [30]. This region of MSH3 is not involved in DNA or nucleotide binding [32] and this MutSβ complex has the same binding affinities for homoduplexes, tailed substrates and insertion/deletion loops (IDLs) as complexes containing the full length MSH3 protein, as well as the same rate constants and ATPase activities on these substrates [30]. Use of this MSH3 variant has the advantage of producing a DNA:protein complex with a mobility that is distinctly different from that of the DNA:MutSα complex. Both MutS complexes were of equivalent concentration as evidenced by the fact that equivalent amounts of protein contained equivalent amounts of MSH2 (S2 Fig, panel A). There was also no evidence of any degradation of the subunits as evidenced by the single products detectable on western blotting with antibodies to MSH2, MSH3 and MSH6 (S2 Fig, panel A).

As expected MutSα does not bind well to either homoduplex DNA or a loop-out of (CA)_3_, a good substrate for MutSβ-mediated but not MutSα-mediated repair (S2 Fig, panel B). In contrast, MutSβ binds effectively to the (CA)_3_ loop-out with even low concentrations of protein being able to shift almost all of the substrate. Limited binding of MutSβ to the homoduplex was also seen (S2 Fig, panel B). This binding is much less extensive than the binding of MutSβ to the (CA)_3_ loop-out as evidence by the fact that no unbound (CA)_3_ substrate was seen at a protein concentration of 0.8 nM, while most of the homoduplex remained unbound even at the highest protein concentration tested (20 nM). Binding of MutSβ to homoduplexes has been previously reported where it has been attributed to end binding [33–35].

**Fig 5:**
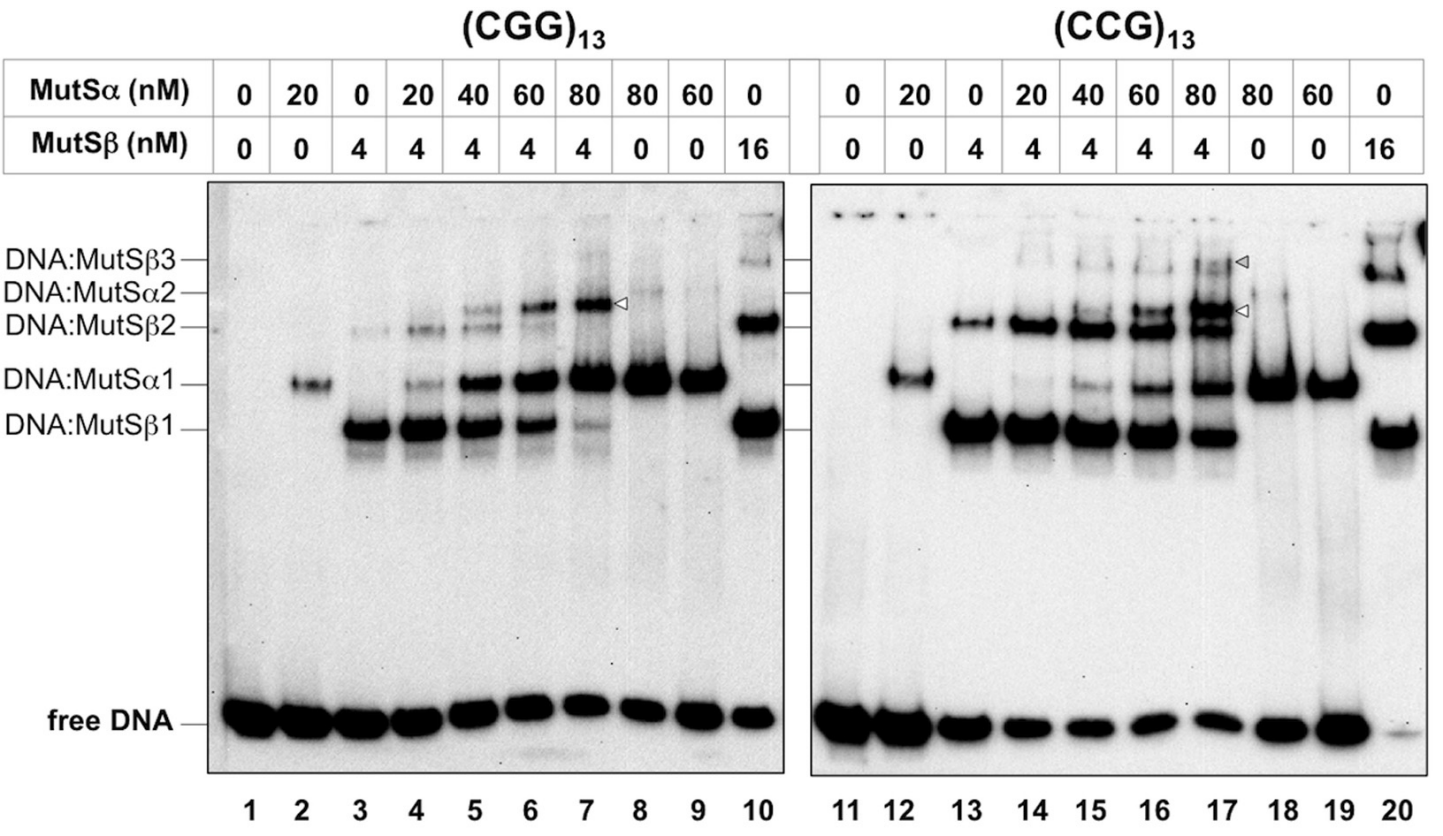
MutSα binds to both strands of the FX repeat and promotes binding of MutSβ. MutSα and/or MutSβ with the N-terminal truncation of MSH3 were added at the indicated concentrations to reaction mixtures containing the CCG repeat and CGG repeat-containing substrates as described in the Materials and Methods. The DNA and DNA:protein complexes were then resolved by native polyacrylamide gel electrophoresis at 4˚C, transferred to nylon membrane and the DNA detected using streptavidin conjugated to horseradish peroxidase (HRP) and a chemiluminescent substrate. The different DNA:MutS complexes are numbered in order of decreasing mobility. The arrowheads indicate novel shifted bands that are not seen in reactions containing either MutSα or MutSβ alone.

MutSα binds to a substrate containing a G•T mismatch (S2 Fig) and to (CCG)_13_ and (CGG)_13_ loop-outs (Fig 5). It also binds to (CAG)_13_ and (CTG)_13_ loop-outs (S2 Fig, panel B). However, MutSα binds less well to the repeat substrates than to the G•T mismatch since binding of MutSα to G•T mismatch depletes all of the free probe at the highest protein concentration, while some free probe remains with all the repeat substrates. MutSα binding to the repeat substrates is also less extensive than the binding of MutSβ (compare lanes 2 and 3 and 12 and 13 of Fig 5).

MutSα stimulates MutSβ binding to a canonical MMR substrate containing a 2 nucleotide insertion/deletion when present at a high MutSα:MutSβ ratio [36]. To test whether this was also true for FX repeat-containing substrates we compared the binding of MutSβ in the presence and absence of an excess of MutSα. MutSβ binding to both canonical and repeat containing substrates produced multiple DNA:protein complexes as can be seen in Fig 5 and S2 Fig. Multiple DNA:MutSβ complexes have been previously reported for both yeast and human proteins binding to canonical MutSβ substrates [34, 35, 37] as well as (CAG)_13_ loop-outs [31]. The different MutSβ containing products could reflect either multiple MutSβ molecules binding to a single DNA molecule or to alternative binding modes. MutSβ binding to (CCG)_13_ and (CGG)_13_ substrates was increased in the presence of MutSα. This was evidenced most clearly as an increase in the amount of the DNA:MutSβ complex with the second highest mobility (DNA:MutSβ2; compare lanes 3 and 4 and 13 and 14 of Fig 5). This is not likely to be a non-specific effect since the addition of much higher concentrations of BSA do not have the same effect (S2 Fig, panel D). The increase in MutSβ binding was associated with a decrease in the amount of the MutSα-shifted band. Since the substrate has not been depleted, this decrease is unlikely to reflect competition between MutSα and MutSβ for binding to the substrate. Rather, it may reflect the incorporation of MutSα into one or more higher molecular weight species. Indeed at higher MutSα concentrations, a new shifted product, indicated by the open arrowheads in Fig 5, is apparent. This product is associated with a decline in the levels of the 2 most rapidly migrating DNA:MutSβ complexes. It is not seen in the absence of MutSβ even when very high concentrations of MutSα are used (lanes 8 and 9 and 18 and 19 of Fig 5). It thus likely represents complexes containing both MutSα and MutSβ. In reactions containing both MutSα and MutSβ a small amount of a second novel band is also seen with the CCG-substrate (indicated by the grey arrowhead in Fig 5, lane 17). This band may represent the result of binding of multiple MutSβ and MutSα complexes to the CCG substrate or complexes in which the binding modes differ from the complex with the faster mobility.

We have previously shown that MutSβ is able to increase the stability of the CCG-loop-out at physiological temperatures. To assess whether MutSα binding had the same effect, we monitored the thermal denaturation of the oligonucleotide in the presence of BSA or MutSα as previously described [14]. Since the 5’ end of the oligonucleotide was labeled with 5-carboxy-X-rhodamine (ROX), a fluorescence donor and the 3’ end was labeled with IOWA Black RQ, a fluorescence acceptor, the stability of the hairpins could be assessed in the presence of protein by monitoring the effect of increasing temperature on the fluorescence at 608 nm, the ROX emission wavelength. The oligonucleotide was denatured and cooled under conditions in which the repeats are known to form hairpins [7, 10, 38–42]. The oligonucleotide was then mixed with either BSA or MutSα and the thermal denaturation of the oligonucleotides monitored as previously described [14]. The melting curves obtained for both protein-CCG-repeat mixtures fit a two-state model (S3 Fig). While the best-fit for CCG-repeat melting in the presence of MutSα was within acceptable limits, the data suggest that the process by which the oligonucleotide melts in the presence of MutSα may be more complex than it is either in the presence of BSA or MutSβ [14]. The thermodynamic parameters derived from analysis of the melting curves are shown in Table 1. As for MutSβ, the presence of MutSα resulted in higher apparent ∆Gs at 37˚C than is seen in the presence of BSA. This suggests that MutSα, like MutSβ, increases the stability of the CCG-repeat structure at physiological temperature. However, the significant differences in the enthalpy of melting (∆H_m_) of the oligonucleotide in the presence of MutSβ (52.4 ± 4.1 kcal/mol; [14]) and MutSα (81.3 ± 3.0 kcal/mol) suggest that the consequence of binding of these two complexes differs. This may reflect the very different modes of binding of these complexes to their substrates [30, 34].

**Table 1:** Comparison of free energy at 37˚C and enthalpy changes required to melt the secondary structure formed by CCG-repeats in the presence of BSA and MutSα.

	∆G (37˚C) kcal/mol	∆H_m_ kcal/mol
**BSA**	1.88±0.08	28.4±0.9
**MutSα**	3.67±0.13	81.3±3.0

## Discussion

We have previously shown that the loss of MSH2 eliminates all germ line and somatic expansions in the FXD mouse and that most of these expansions are MutSβ-dependent since the loss of MSH3 eliminates almost 98% of germ line expansions and all somatic ones [3, 14]. The remaining 2% of MSH2-dependent germ line expansions are presumably the result of MutSα-dependent events. However, we show here that loss of MSH6, and thus MutSα, reduces both the germ line and the somatic expansion frequency by much more than 2% (Figs 1–3).

A comparison of the relative levels of MSH2 in *Msh3*^*-/-*^ and *Msh6*^*-/-*^ mice showed that the loss of MSH6 resulted in a greater decrease of MSH2 than the loss of MSH3 (Fig 4). The fact that no MSH3 or MSH6 is seen in *Msh2*^-/-^ mice (Fig 4) is consistent with previous reports suggesting that the levels of MSH2, MSH3 and MSH6 are interdependent and that there is very little free MSH3 or MSH6 in cells [24, 25]. Thus our data would be consistent with the interpretation that more MSH2 is in a heterodimer with MSH6 than is in a heterodimer with MSH3, *i*.*e*., that MutSα is more abundant than MutSβ in these animals. This finding is consistent with what has been reported for human cells [43, 44] and mice with a mixed C57BL6/129/OLA/FVB background [16], but not with what is seen in FVB mice [28]. While we did not assess the absolute amount of MSH3, a comparison of the relative levels of MSH3 in *Msh6*^*+/+*^ and *Msh6*^*-/-*^ mice showed that the loss of MSH6 did not result in a detectable decrease in MSH3 levels in expansion-prone organs like brain and testes (Fig 4). Since MSH2 levels were reduced in *Msh6*^*-/-*^ mice it is possible that MSH6 is acting indirectly via decreasing the amount of MSH2 available to form the MutSβ complex. However, since MSH3 levels are not significantly lower in *Msh6*^*-/-*^ mice and MSH3 is thought to be stable only when in the MutSβ complex [24, 25], MSH6 may well be playing a different role in the expansion process.

No comparable decrease in expansions is seen in *Msh6*^*-/-*^ mice in models of other repeat expansion diseases where MSH3 has been implicated in the expansion process and where a similar excess of MutSα was seen [16]. In addition, the loss of MSH6 in the same mouse strain or in human cells does not result in reduced repair of typical MutSβ substrates [45–49]. Thus significant redeployment of MutSβ to other sites in the genome to compensate for the loss of MutSα is also unlikely to account for the decrease in germ line and somatic expansions seen in *Msh6*^*-/-*^ mice. However, since very few germ line expansions and no somatic ones are detected in mice that lack MutSβ [14], MutSα is not able to efficiently substitute for MutSβ in the expansion process despite the relative abundance of MutSα in these animals (Fig 4). A contribution by both MutSα and MutSβ to repeat expansion is consistent with our previous observations that while MutSβ levels alone do not correlate well with the levels of somatic instability across 5 different organs, a better correlation is seen when the levels of both MutSβ and MutSα are considered [3].

Thus, our data suggest that the involvement of MutSα in repeat expansion is not unique to somatic expansion of GAA/TTC-repeats in FRDA, contributing to both germ line and somatic expansion in the FXD mouse. Whether MutSα acts independently of MutSβ in FRDA unknown. The nature of MutSα’s role in the expansion process in the FXD mouse is also unclear. MutSα may be acting to facilitate MutSβ-dependent expansion by increasing the stability of the expansion substrates as it does *in vitro* (Table 1), or by protecting the substrates from repair by another mechanism, thus allowing more time for the hairpins or other atypical structures to be processed by MutSβ to generate expansions. MutSα also promotes binding of MutSβ to the repeat substrates (Fig 5) in a manner reminiscent of MutSα’s effect on MutSβ binding to a canonical MutSβ substrate [36]. This property may reflect yet another way that MutSα could facilitate MutSβ-mediated repeat expansions.

A role for MutSα in repeat expansion has not been observed in mouse models of CTG/CAG-repeat expansion diseases despite the fact we have shown that MutSα binds to those repeats *in vitro* (S2 Fig). It may be that the effect of the loss of MutSα is only apparent under certain circumstances. For example, it may be that in the FXD mouse model where the expansion frequency is high, the amount of the expansion substrate formed in the germ line exceeds the processing capacity of MutSβ acting alone. Under these conditions the effect of MutSα would become apparent. When only moderate levels of the expansion substrate are produced, an effect of the loss of MutSα might only be seen in mice with reduced MutSβ levels since the available MutSβ in *Msh3*^*+/+*^ animals may be sufficient to process all the expansion substrates without the assistance of MutSα. At the other end of the spectrum when the expansion substrates are present only at very low levels, either one of the MutS complexes may be sufficient to process them. This may explain the perplexing observation that in a mouse model of Huntington disease, loss of MSH2 eliminates germ line expansions but neither the loss of MSH3 nor the loss of MSH6 had any effect on the expansion frequency [18]. This idea would also be consistent with our observation that in the FXD mouse, the loss of MSH6 had more of an effect on somatic expansion in males than in females (Fig 1), since the expansion process is less extensive in females [21] and thus the requirement for MutS proteins may be lower. Thus, the different effects of MutSα and MutSβ on expansion in different models, in germ line versus somatic cells, or in males and females, may not necessarily reflect differences in the mechanisms of expansion, but rather differences in the levels of the MutS complexes relative to the substrates that potentially could be processed to generate expansions.

In addition to contributing to the generation of expansions, our data suggest that MutSα may also act to protect against germ line contractions as evidenced by the reduction in the proportion of unchanged alleles in *Msh2*^-/-^ [23] and *Msh6*^*-/-*^ animals (Fig 2) relative to *Msh3*^*-/-*^ mice [14]. Protection against germ line contractions by MutSα would be consistent with what has been reported for both the GAA/TTC-mouse model [17] and a CAG/CTG-mouse model [18]. Protection against contractions by MutSα also would be consistent with a typical MMR process albeit one that is triggered by an atypical repair substrate. The nature of the FX hairpins with the high frequency of single mismatches may account for the ability of MutSα to bind to and coordinate their repair. The ability of MutSα to contribute both to error-free repair and to expansions may reflect MutSα’s ability to participate in more than one DNA repair pathway [50, 51].

We have recently demonstrated that a hypomorphic mutation in Polβ, a key DNA polymerase involved in base excision repair (BER), reduces expansion in the FXD mouse [52]. How MutSα and MutSβ interface with the BER pathway to generate expansions in these models remains an open question. One possibility is that MutSβ and MutSα act downstream of DNA damage excision to stabilize loop-outs formed during strand-slippage and strand-displacement synthesis that is mediated at least in part by Polβ. We speculate that normal signaling by MutSα results in MMR of these loop-outs resulting in error-free repair, while MutSβ, alone or together with MutSα, can channel them into a different repair pathway that results in expansions.

## Materials and Methods

### Ethics statement

This work was carried according to ARAC guidelines and procedures as outlined in the Guide for the Care and Use of Laboratory Animals, U.S. Government Principles for the Utilization and Care of Vertebrate Animals Used in Testing, Research, and Training and Public Health Service Policy on Humane Care and Use of Laboratory Animals. This work was approved by the NIDDK Animal Care and Use Committee (ASP: K021-LMCB-12 and K021-LMCB-15).

### Oligonucleotides and proteins

Oligonucleotides were obtained from Integrated DNA technologies (IDT, Coralville IA) and are listed in Table 2. Purified human MutSα was a kind gift of Drs Hsieh and Geng (NIDDK, NIH). Purified human MutSβ was a kind gift of Drs Yang and Li (NIDDK, NIH). This MutSβ complex contained a “trimmed” version of MSH3 containing amino acids 211–1125. This MutSβ complex has the same binding affinities for homoduplexes, tailed substrates and IDLs as complexes containing the full length MSH3 protein, as well as the same rate constants and ATPase activities [30].

**Table 2:** Oligonucleotides used in this study.

Name	Sequence	Assay
**M010**	5'-TGGAAGGATTGGAGCTACGG-3'	MSH6 genotyping
**M011**	5'-TTACCTCCTCCACTGACGTG-3'	MSH6 genotyping
**M012**	5'-CAAGCCCCTTTCTTTGTTTG-3'	MSH6 genotyping
**M013**	5'-ACCACTTCCTCATCCCTGG-3'	MSH6 genotyping
**FraxM4**	5'-FAM-CTTGAGGCCCAGCCGCCGTCGGCC-3'	FX genotyping
**FraxM5**	5'-CGGGGGGCGTGCGGTAACGGCCCAA-3'	FX genotyping
**FraxC**	5'-GCTCAGCTCCGTTTCGGTTTCACTTCCGGT-3'	FX genotyping
**FraxF**	5'-AGCCCCGCACTTCCACCACCAGCTCCTCCA-3'	FX genotyping
DuplexBS*	5'-CTGCCTCAAGTGTTCGGACTCTGCCTCAAATGACGGTAGTCAACGTGCTTGGACGGTAGT-3'	EMSA
**DuplexTS**	5'-ACTACCGTCCAAGCACGTTGACTACCGTCATTTGAGGCAGAGTCCGAACACTTGAGGCAG-3'	EMSA
**(CNG)**_**13**_TS**	5'-ACTACCGTCCAAGCACGTTGACTACCGTCACNGCNGCNGCNGCNGCNGCNGCNGCNGCNGCNGCNGCNGTTTGAGGCAGAGTCCGAACACTTGAGGCAG-3'	EMSA
**(CA)**_**3**_**TS**	5'-ACTACCGTCCAAGCACGTTGACTACCGTCACACACATTTGAGGCAGAGTCCGAACACTTGAGGCAG-3'	EMSA
**GT-TS**	5'-ACTACCGTCCAAGCACGTTGACTACCGTC**G**TTTGAGGCAGAGTCCGAACACTTGAGGCAG-3'	EMSA
**(CCG)**_**10**_	5'-ROX-(CCG)_10_-IOWA Black RQ-3'	Thermal melting

*This oligonucleotide was labeled at the 5’ end with biotin during synthesis for use in EMSA reactions.

**N represents either A, G, C or T.

### Mouse maintenance

The generation of the FXD mice was described previously [2]. These mice are on a C57BL/6 background. The *Msh6*^*+/-*^ mice were generated previously [49, 53] and cryopreserved embryos were obtained from the NCI Mouse Repository (Frederick, MD). These mice are also on a predominantly C57BL/6 background. Live born pups were generated from these embryos by implantation into the oviduct of pseudopregnant recipients using standard procedures. F2 *Msh6*^+/-^ parents were bred to generate *Msh6*^*+/+*^, *Msh6*^*+/-*^ and *Msh6*^*-/-*^ littermates. Multiple breeding pairs from the same parents were set up for each genotype. The litters for each genotype considered for this analysis had a similar parental age distribution. This was the same genetic background and breeding strategy that we had used previously to examine the effect of the loss of MSH3 on the expansion frequency [14]. Mice were maintained in accordance with the guidelines of the NIDDK Animal Care and Use Committee and with the *Guide for the Care and Use of Laboratory Animals* (NIH publication no. 85–23, revised 1996).

### Genotyping and analysis of repeat number

Sperm was isolated from the cauda epididymis as previously described [54], pelleted twice by centrifugation at 500 g for 5 min and the pellet resuspended first in PBS and then in 100 μl of a solution containing a 90:10 mixture of ATL lysis buffer (Qiagen, Valencia, CA) and a 20 mg/ml proteinase K solution (Invitrogen, Carlsbad, CA). The samples were then incubated at 55°C overnight before the addition of 30 μl of 5 M NaCl. The resultant precipitate was pelleted by centrifugation and the supernatant transferred to a new tube and mixed with 130 μl of ethanol. The DNA was then pelleted by centrifugation and dissolved in TE by incubation overnight at 55°C. This protocol results in little, if any, contamination with somatic DNA [54]. Genomic DNA from mouse tails was extracted using KAPA Mouse Genotyping Kit (KAPA Biosystems, Wilmington, MA). Genomic DNA from other tissues was extracted using a Maxwell16 Mouse tail DNA purification kit (Promega, Madison, WI) according to the manufacturer’s instructions. *Msh6* genotyping was carried out with Taq DNA polymerase in standard buffer with either the M010/M011 primer pair to detect the WT allele and M012/M013 to detect the mutant allele. The PCR parameters were 1x 94°C for 1 min., 35x (94°C for 1 min., 60°C for 2 min. and 72°C for 1 min), followed by 1x 72°C for 3 min. The presence of the PM allele and its repeat number was determined using a fluorescent PCR assay and FraxM4 and FraxM5 primer pair as described previously [3]. The somatic instability index (SII) was calculated from the GeneMapper profiles of DNA from different organs as previously described [3, 22] and used to evaluate the extent of somatic expansion in adult mice. For small pool PCR analysis from sperm, the DNA was diluted to 3 pg/μl (roughly 1 haploid genome equivalent/μl). The diluted DNA was then subjected to nested PCR. The first round of PCR was carried out using the primers FraxC and FraxF in a 25 μl PCR mix as described previously [55]. One microliter of this PCR mix was used in second round of PCR with the FraxM4 and FraxM5 primers. Roughly 50% of the reactions contained a PCR product, consistent with the idea that each positive PCR likely represents the products of amplification of DNA from a single sperm cell. An exact Jonckheere-Terpstra test of trend in ordered counts was carried out using StatXact software (version 8; Cambridge, Massachusetts). Fisher’s exact test was carried out using the GraphPad QuickCalcs website (http://www.graphpad.com/quickcalcs). The Mann-Whitney *U* test was carried out using VassarStats (http://vassarstats.net/). We set the significance level (α) at 0.050 for the pairwise comparisons. For the comparisons of WT, heterozygous and homozygous null animals this corresponds to p = 0.015 after adjusting for multiple testing using the (relatively conservative) Bonferroni correction. Hartigans’ dip test was calculated using the dip.test command in the R diptest library.

### Western blotting

Total protein extracts were prepared from flash frozen brain, liver, testes and ovary of 6-month old mice. Tissues were homogenized using a tissue homogenizer (Precellys 24, Bertin Technologies, Berlin, Germany) with T-PER protein extraction reagent (Pierce Biotechnology, Inc, Rockford, IL) supplemented with complete, Mini, EDTA-free protease inhibitor cocktail (Roche Applied Science, Indianapolis, IN). Nuclear extracts of liver proteins were prepared using the NE-PER Nuclear and cytoplasmic extraction reagents (Pierce Biotechnology, Inc, Rockford, IL) according to the manufacturer’s instructions. The protein concentrations were determined using a Bio-Rad protein assay kit (Bio-Rad, Hercules, CA). Proteins were heated for 10 minutes at 70°C in LDS-Sample Buffer (Life Technologies, Grand Island, NY), resolved by electrophoresis on either 3–8% NuPAGE Novex Tris-Acetate gels (Life Technologies) or 4–12% NuPAGE Novex Tris-Bis gels (Life Technologies) and transferred to nitrocellulose membranes using the iBlot transfer apparatus (Life Technologies) according to the manufacturer’s instructions. Membranes were blocked for one hour at room temperature in 5% ECL Prime blocking agent (GE Healthcare Bio-Sciences) in TBST, then incubated overnight at 4°C with antibodies to MSH2 (ab70270, Abcam, Cambridge, MA) at a concentration of (1:10000), MSH3 (sc-271079, Santa Cruz, Dallas, TX) at a concentration of (1:1000) and MSH6 (BD 610918, BD Biosciences, Franklin Lakes, NJ) at a concentration of (1:1000). The secondary antibodies (anti-mouse IgG, NA931V and anti-rabbit IgG, NA934V, GE Healthcare Bio-Sciences) were both used at a dilution of 1:5000. After addition of the ECL Prime detection reagent (GE Healthcare Bio-Sciences), the blot was imaged using a Fluorchem M imaging system (Proteinsimple, Santa Clara, CA). Beta-actin (anti-mouse ab8227, Abcam, Cambridge, MA) was used as a loading control for total cell extracts and lamin B (ab16048, Abcam, Cambridge, MA) for nuclear extracts. A representative example of a full blot of testes protein extracts showing binding to MSH2, MSH3, MSH6 and the loading control β-actin is shown in S1 Fig. Western blots were repeated several times and always included molecular weight markers and extracts from the appropriate null mice as negative controls. To evaluate whether the loss of MSH6 affected the levels of MSH3 in *Msh6*^*-/-*^ mice, knowledge of the absolute levels of each protein is not necessary. Since the levels of MSH3 in each group of animals was tested with equivalent amounts of protein using the same antibody on the same gel, the avidity of the MSH3 antibody relative to the avidity of the MSH6 or MSH2 antibodies is not an issue. We thus were able to directly compare the levels of MSH3 in WT, *Msh3*^*-/-*^ and *Msh6*^*-/-*^ animals by determining the amount of each protein relative to β-actin (total protein extracts) or lamin B1 (nuclear extracts) using the AlphaView software for FluorChem Systems (Proteinsimple, Santa Clara, CA). The levels of MSH2 and MSH6 in these animals were determined in the same way.

### EMSA analysis of MutSα binding to the CGG- and CCG- loop-outs

The oligonucleotides used in EMSA were prepared as described previously [14]. The binding reactions were carried out using the Gelshift chemiluminescent EMSA kit (Active Motif, Carlsbad, CA) according to the manufacturer’s instructions using the indicated amounts of purified human MutSα and human MutSβ and 2 fmoles of the duplexed oligonucleotides as described previously [14].

### Thermal analysis of the CCG-repeats in the presence of MutSα

The oligonucleotide used for thermal analysis consisted of a single strand of DNA comprised of 10 copies of CCG with the 5’ end labeled with 5-carboxy-X-rhodamine (ROX) and the 3’ end with IOWA Black RQ. The oligonucleotide was prepared as described previously [14] and MutSα or BSA was added to 360 nM as indicated. Thermal denaturation was monitored as described previously [14]. The melting curve was consistent with a two-state model (S3 Fig) and the thermodynamic parameters were thus derived from the melting curve using a two-state model (closed and open states).

## Supporting Information

S1 Fig Representative western blot showing simultaneous reaction of protein extracts from WT, *Msh3*^*-/-*^ *and Msh6*^*-/-*^ mouse testes with antibodies to MSH2, MSH3 and MSH6. Three different animals of each genotype are shown. The *Msh2*^-/-^ sample represents the same amount of protein derived from pooling testes extracts from 3 different males. This sample serves as a negative control for the 3 antibodies since these mice lack MSH2, MSH3 and MSH6. The normalization control, β-actin is also shown on the same blot. A much lower exposure of this gel was used for the β-actin quantitation. The bands indicated by the asterisk represent non-specific products resulting from the use of the MSH3 mouse monoclonal antibody.
(TIFF)

S2 Fig MutSα and MutSβ binding to canonical and noncanonical substrates. A) Quantitation of MutSα and MutSβ. Equimolar amounts of MutSα and MutSβ based on protein concentration were resolved by SDS-PAGE. The resolved proteins were transferred to nitrocellulose membrane and challenged with antibodies to MSH2, MSH3 and MSH6. The similarity in the intensity of the MSH2-reacting products in the MutSα and MutSβ lanes confirms that they contain very similar amounts of MSH2 and thus comparable concentrations of protein reflect comparable levels of MutSα and MutSβ. B) and C) Different amounts of MutSα and MutSβ were added to reaction mixtures containing either a fully homoduplex molecule or otherwise duplex oligonucleotides containing the indicated mismatched or IDL substrates as described in the Materials and Methods. For the G•T substrate 0.8, 4 and 20 nM of each protein was used. For the remaining substrates 0.16, 0.8, 4, and 20 nM of each protein was used. The DNA and DNA:MutS complexes were then resolved by native polyacrylamide gel electrophoresis at 4°C, transferred to nylon membrane and the DNA detected using streptavidin conjugated to horseradish peroxidase (HRP) and a chemiluminescent substrate. Note that the same molar concentration of substrate and similar exposures were used throughout. The higher signal coming from the free probe in panel B, may reflect the fact that these substrates are smaller than the substrates shown in panel C, and thus may be transferred more efficiently to the nylon membrane. However, since the free probes used in panel C are all the same size, it is possible to compare the extent of binding to the substrates for the different repeat-containing probes. D) (CGG)_13_ and (CCG)_13_ were incubated with MutSβ along with the indicated amounts of BSA.
(TIFF)

S3 Fig Melting of the CCG-repeat in the presence of MutSα fits a two-state model. A and B). Melting curves produced on thermal denaturation of a (CCG)_10_ oligonucleotide labeled at the 5’ end with ROX and at the 3’ end with IOWA Black RQ. The intensity of fluorescence of the ROX donor was plotted against temperature in the presence of BSA (A) or MutSα. Dots represent the experimental data and solid lines are the best-fits according to the two-state model. The panels on the right show the distribution of residuals.
(TIFF)
