# Supplementary figures and images for: A MutSβ-Dependent Contribution of MutSα to Repeat Expansions in Fragile X Premutation Mice?
Source: PLoS Genet. 2016 Jul 18;12(7):e1006190. doi: 10.1371/journal.pgen.1006190 (PMC4948851; doi:10.1371/journal.pgen.1006190)

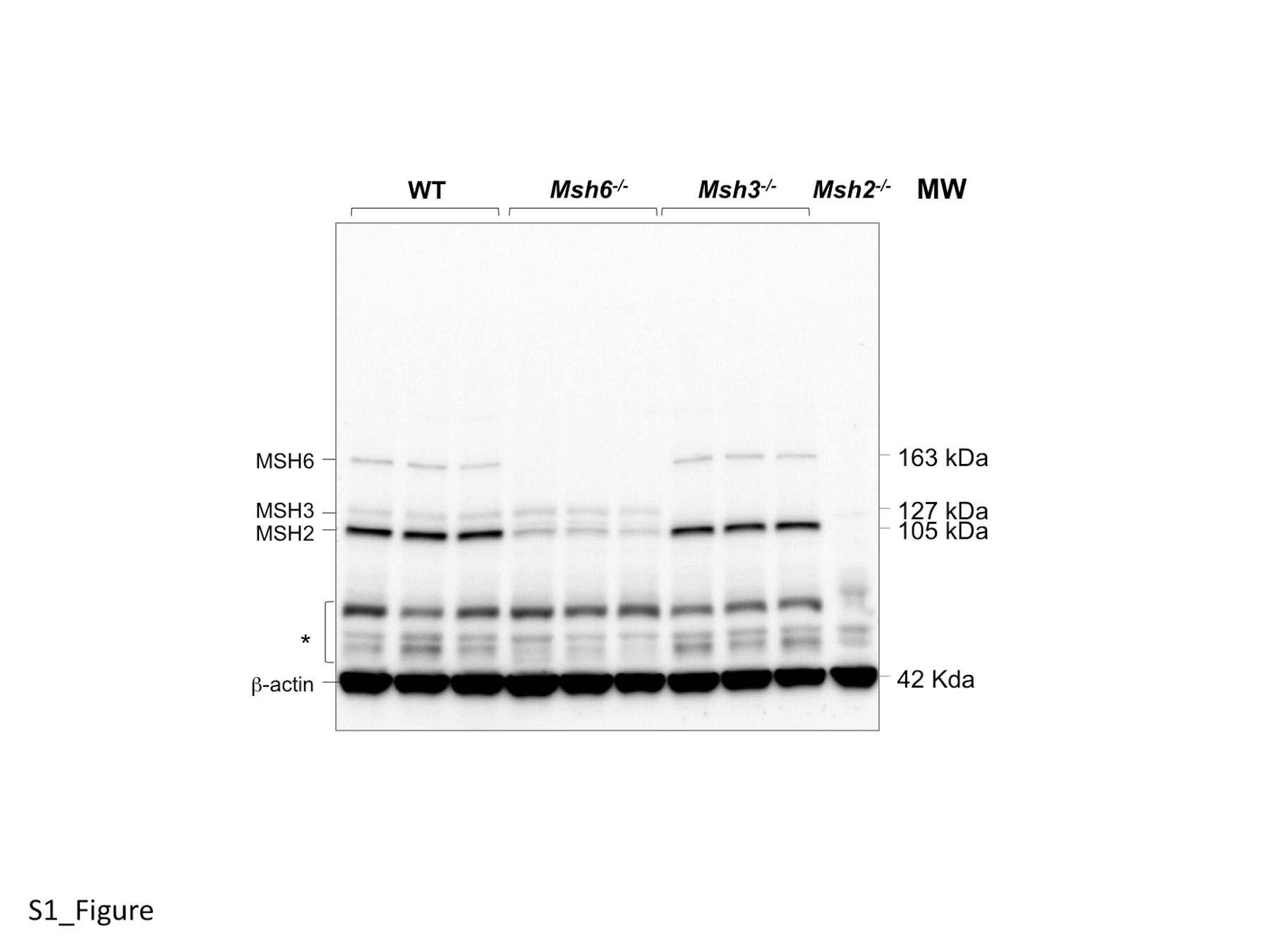

Supplement: S1 Fig — Three different animals of each genotype are shown. The Msh2-/- sample represents the same amount of protein derived from pooling testes extracts from 3 different males. This sample serves as a negative control for the 3 antibodies since these mice lack MSH2, MSH3 and MSH6. The normalization control, β-actin is also shown on the same blot. A much lower exposure of this gel was used for the β-actin quantitation. The bands indicated by the asterisk represent non-specific products resulting from the use of the MSH3 mouse monoclonal antibody. (TIFF) [file pgen.1006190.s001.tiff]

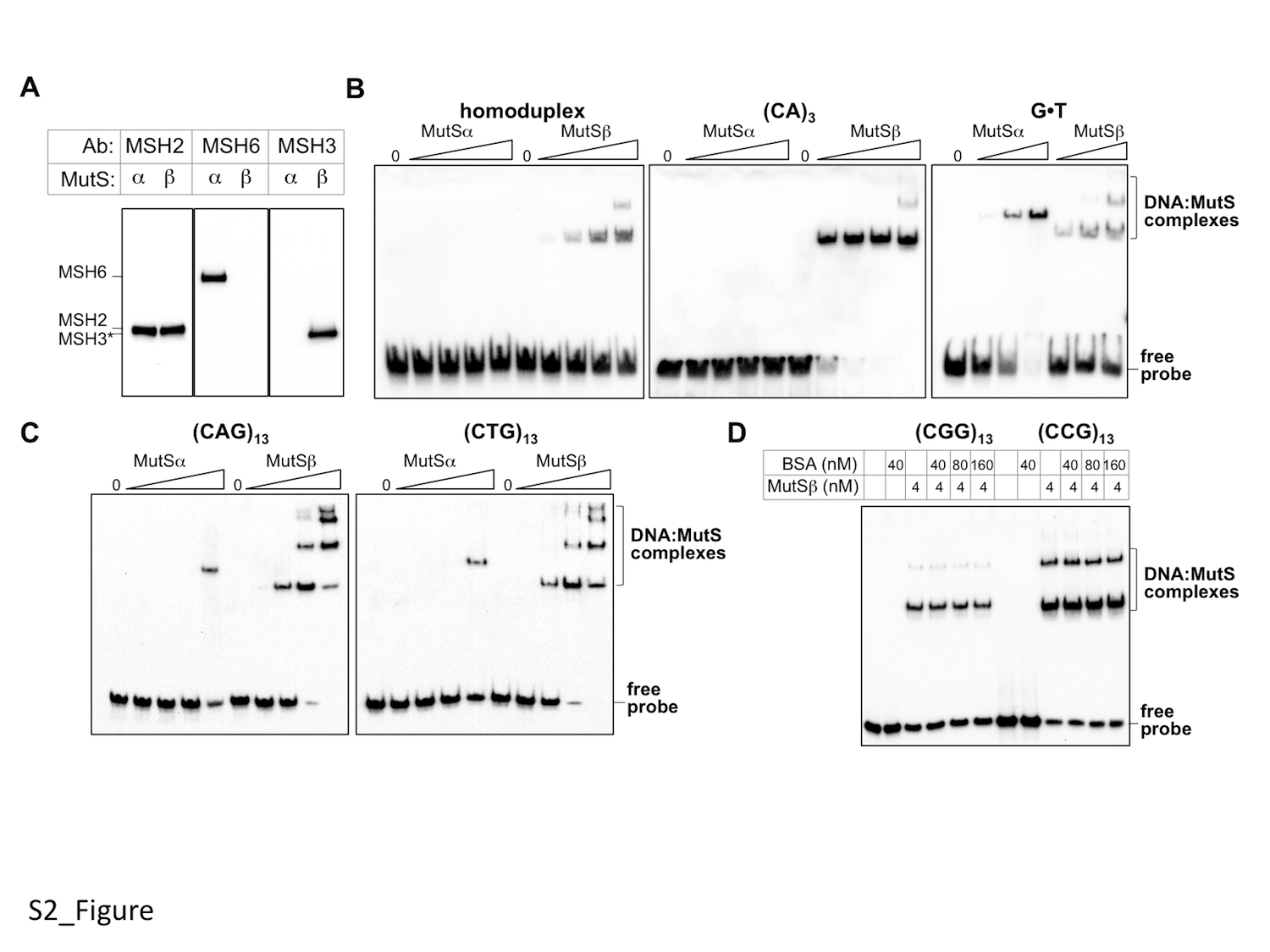

Supplement: S2 Fig — A) Quantitation of MutSα and MutSβ. Equimolar amounts of MutSα and MutSβ based on protein concentration were resolved by SDS-PAGE. The resolved proteins were transferred to nitrocellulose membrane and challenged with antibodies to MSH2, MSH3 and MSH6. The similarity in the intensity of the MSH2-reacting products in the MutSα and MutSβ lanes confirms that they contain very similar amounts of MSH2 and thus comparable concentrations of protein reflect comparable levels of MutSα and MutSβ. B) and C) Different amounts of MutSα and MutSβ were added to reaction mixtures containing either a fully homoduplex molecule or otherwise duplex oligonucleotides containing the indicated mismatched or IDL substrates as described in the Materials and Methods. For the G•T substrate 0.8, 4 and 20 nM of each protein was used. For the remaining substrates 0.16, 0.8, 4, and 20 nM of each protein was used. The DNA and DNA:MutS complexes were then resolved by native polyacrylamide gel electrophoresis at 4°C, transferred to nylon membrane and the DNA detected using streptavidin conjugated to horseradish peroxidase (HRP) and a chemiluminescent substrate. Note that the same molar concentration of substrate and similar exposures were used throughout. The higher signal coming from the free probe in panel B, may reflect the fact that these substrates are smaller than the substrates shown in panel C, and thus may be transferred more efficiently to the nylon membrane. However, since the free probes used in panel C are all the same size, it is possible to compare the extent of binding to the substrates for the different repeat-containing probes. D) (CGG)13 and (CCG)13 were incubated with MutSβ along with the indicated amounts of BSA. (TIFF) [file pgen.1006190.s002.tiff]

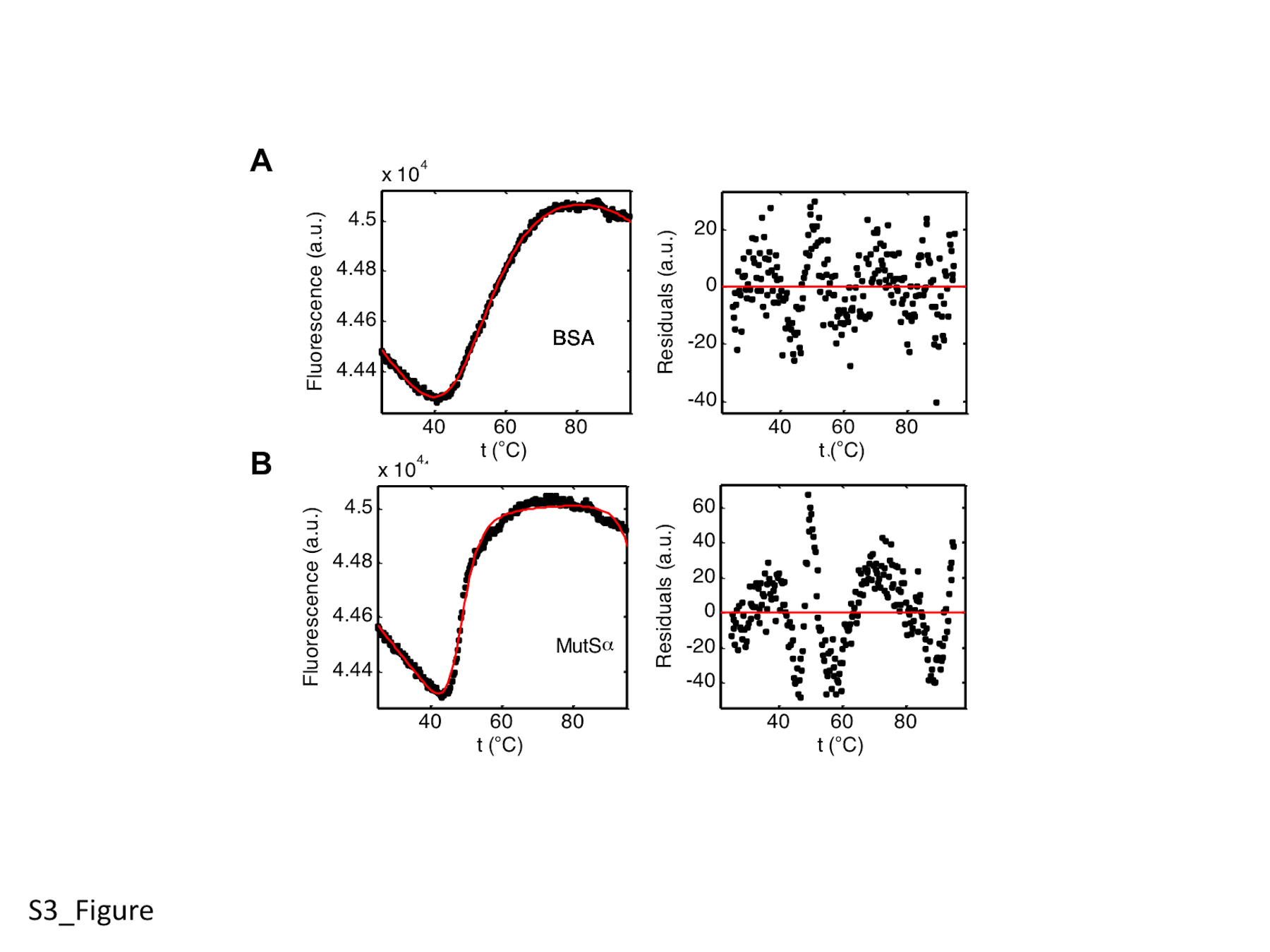

Supplement: S3 Fig — A and B). Melting curves produced on thermal denaturation of a (CCG)10 oligonucleotide labeled at the 5’ end with ROX and at the 3’ end with IOWA Black RQ. The intensity of fluorescence of the ROX donor was plotted against temperature in the presence of BSA (A) or MutSα. Dots represent the experimental data and solid lines are the best-fits according to the two-state model. The panels on the right show the distribution of residuals. (TIFF) [file pgen.1006190.s003.tiff]
